# Clinical Equivalence of a CNN-Based Automated Soft Tissue Landmark Detection System on 2D Facial Images

**DOI:** 10.3390/diagnostics16101464

**Published:** 2026-05-11

**Authors:** Argun Ege Türkün, Müslim Ege Kalender, Murat Kurt, Servet Doğan

**Affiliations:** 1 Department of Orthodontics, Faculty of Dentistry, Ege University, 35040 Izmir, Turkey; servetdgn@hotmail.com; 2 International Computer Institute, Department of Computer Science, Ege University, 35040 Izmir, Turkey; ege.kalender45@gmail.com (M.E.K.); murat.kurt@ege.edu.tr (M.K.)

**Keywords:** orthodontics, facial soft tissue landmark, deep learning, convolutional neural network, 2D facial imaging, automated landmark detection, artificial intelligence, digital workflow

## Abstract

**Background/Objectives:** The aim of this study was to evaluate and compare the accuracy, reliability, and time efficiency of a convolutional neural network (CNN)-based deep learning model with manual annotation in the identification of soft tissue landmarks on two-dimensional (2D) facial images for orthodontic applications. **Materials and Methods:** Three-dimensional (3D) facial scans were obtained from 100 participants (50 females, 50 males) aged 18–25 years using the Revopoint Pop2 3D Scanner. Frontal and profile 2D images were extracted from the 3D models. Manual landmark identification was performed by a single investigator using LabelMe software, marking 22 landmarks on frontal images and 15 landmarks on profile images. A novel CNN model was developed and trained on these manually annotated images. The model’s automatic landmark identifications were compared with manual annotations in terms of positional error, identification time, and reproducibility. **Results:** The CNN model achieved a mean localization accuracy of 96.07%. The mean prediction error ranged from 2.3% to 4.5% across various anatomical points. Trichion, Menton, and Gonion points exhibited relatively higher error rates. The model significantly reduced the annotation time compared to manual identification (manual method: 237 s per image). Intra-observer reliability analysis demonstrated excellent agreement for manual landmarking (ICC: 0.85–0.95). The AI model provided consistent predictions for identical inputs. **Conclusions:** The deep learning-based model demonstrated comparable accuracy to manual landmark identification while significantly improving the annotation speed and reproducibility. These results suggest that CNN-based systems offer a promising alternative for clinical orthodontic analysis and digital workflow integration.

## 1. Introduction

Orthodontic treatment extends beyond tooth alignment to encompass the aesthetics and functional harmony of the face. Accordingly, accurate assessment of soft tissue morphology is a cornerstone of individualized diagnosis and treatment planning. Manual identification of facial soft-tissue landmarks—still considered the practical gold standard—can be time-consuming, labor-intensive, and subject to inter- and intra-observer variability, especially for landmarks with ambiguous anatomical contours such as Gonion, Menton, or Trichion [1,2,3]. These limitations have motivated research into automated, reproducible pipelines that maintain clinical interpretability while reducing workload.

Recent advances in artificial intelligence (AI) and deep learning (DL) have transformed medical imaging, including segmentation, measurement, and landmark localization tasks [4]. In facial soft-tissue analysis, CNN-based methods have achieved significant performance gains over traditional statistical models [5]. In orthodontics, DL applications have expanded rapidly across 2D facial photographs, lateral cephalograms, and 3D imaging modalities [6,7,8,9,10]. Systematic reviews and meta-analyses report mm-level mean absolute errors (MAEs) and high success rates within 2–4 mm error thresholds (SR@2–4 mm), underscoring the potential clinical applicability of these methods [11,12,13,14].

While most studies employ pretrained architectures (e.g., ResNet, EfficientNet) originally designed for natural image classification, their suitability for millimetric-precision facial landmark detection remains an empirical question. Custom CNN architectures tailored specifically for landmark localization may offer competitive or superior performance with reduced computational demands. Systematic comparison of custom designs versus transfer learning approaches is necessary to guide architecture selection for clinical orthodontic applications.

On 2D non-ionizing facial photographs, DL systems can localize dozens of landmarks and compute anthropometric measures with reliability comparable to manual methods while substantially reducing annotation time [2]. On 3D facial images, patch-based and encoder–decoder CNNs have demonstrated accurate soft-tissue landmark localization and segmentation in diverse populations [15,16]. On radiographs/CBCT, meta-analyses indicate steadily improving mm-scale errors and SR@2–4 mm rates, although reporting standards and data heterogeneity remain concerns [12,13,14].

Despite this progress, key methodological gaps remain. First, many studies report errors in pixels, without converting to millimeters, limiting clinical interpretability [5,11]. Second, there is inconsistent transparency in reporting architectures, hyperparameters, data splits, augmentation pipelines, and availability of code or weights [17,18]. Third, very few studies apply formal clinical equivalence testing between AI-based and manual landmarking [19,20,21,22]. Without these elements, robust clinical translation is difficult, especially for orthodontic applications where millimetric precision matters. Orthodontic treatment planning requires millimetric precision; pixel-based errors that appear numerically small often exceed clinical thresholds (2 mm) after conversion. Additionally, statistical significance does not ensure clinical equivalence. Formal equivalence testing (e.g., TOST with ±2 mm margins) is necessary but remains underutilized in AI-based landmark detection.

To address these challenges, recent reporting guidelines such as TRIPOD+AI, CLAIM, and STARD-AI have been developed to improve reproducibility and facilitate clinical adoption of AI models [23,24,25,26]. These frameworks emphasize subject-wise data splitting to prevent identity leakage and complete method transparency, reporting clinically meaningful metrics (e.g., SR@2–4 mm, CED/AUC-CED) with uncertainty intervals and using appropriate statistical methods to assess agreement and equivalence [23,24,25,26].

Few studies have systematically integrated mm-based calibration, formal clinical equivalence testing (TOST), and architectural comparison (custom CNN vs. transfer learning) within a unified framework. This study addresses the above gaps in three keyways. First, we applied per-subject pixel-to- millimeter calibration using 3D-to-2D scaling to express all errors in clinically interpretable units, along with SR@2/3/4 mm and cumulative error distribution (CED) analyses. Second, we ensured full methodological transparency by detailing our CNN architecture (encoder–decoder heatmap regression), training hyperparameters, augmentation scheme, and performing subject-wise data splitting with 95% confidence intervals. Third, instead of relying solely on null-hypothesis significance testing, we conducted two one-sided tests (TOST) for equivalence with a prespecified ±2 mm margin, complemented by Bland–Altman analysis of agreement and limits-of-agreement [20,27,28]. We hypothesize that—after mm calibration—our CNN model will be clinically equivalent to expert manual landmarking for most soft-tissue points, while substantially reducing the annotation time.

## 2. Materials and Methods

This study was designed as a cross-sectional methodological research project aiming to compare manual and artificial intelligence-based methods for soft tissue landmark identification.

This study was approved by the Clinical Research Ethics Committee of Ege University Faculty of Medicine (Approval No: 25-3.1T/79; Date: 20 March 2025) and was conducted in accordance with the Declaration of Helsinki (1975, revised in 2013).

Written informed consent was obtained from all participants prior to inclusion in the study.

The study included 100 individuals (50 females and 50 males) who applied to the orthodontics department of a dental faculty for orthodontic treatment and met the specific selection criteria. All participants were from a Turkish population and were recruited from a single-center orthodontic clinic, reflecting a relatively homogeneous demographic and morphological background. Inclusion and exclusion criteria were applied to the selection of participants.

The inclusion criteria were as follows: individuals between the ages of 18 and 25 years; no history of surgical intervention or trauma affecting the facial structures; no prior orthodontic treatment; and no systemic diseases affecting general health. Additionally, participants were required to possess complete facial images and to voluntarily agree to participate in the study by signing the informed consent form.

The exclusion criteria were having congenital facial anomalies (such as cleft lip and palate or micrognathia); a history of surgical intervention in the facial region (such as orthognathic surgery); a history of previous orthodontic treatment; or systemic diseases affecting facial muscles or bone structures. Furthermore, participants with low-quality scan data, incomplete, missing, or distorted images were excluded from the study.

High-resolution three-dimensional (3D) facial scans were obtained using the Revopoint Pop2 3D Scanner (Revopoint, Shenzhen, China). The scans were performed in a natural head position with eyes closed, ensuring that the complete anatomical structure of the face was captured.

The data obtained from the scans were converted into two-dimensional (2D) projections in the frontal and sagittal planes. These 2D images were used for soft tissue landmark identification through two different methods.

The first method, manual landmarking, was performed by the researcher using the open source LabelMe software (version 5.0, MIT Computer Science and Artificial Intelligence Laboratory, Cambridge, MA, USA). To evaluate intra-observer measurement error, the same investigator repeated the landmark annotations on a randomly selected subset of images (*n* = 20) after a two-week interval. The agreement between the first and second measurements was assessed using the intraclass correlation coefficient (ICC).

During the annotation process, 22 anatomical landmarks were identified on the frontal images and 15 anatomical landmarks on the profile images.

The identified landmarks were as follows: Trichion, Glabella, Nasion, Pronasale, Columella, Subnasale, Labium Superior, Stomion, Labium Inferior, Menton, Pogonion, Gnathion, Gonion (left and right), Chelion (left and right), Endocanthion (left and right), Exocanthion (left and right), Alare (left and right), and Philtrum.

**Figure 1:**
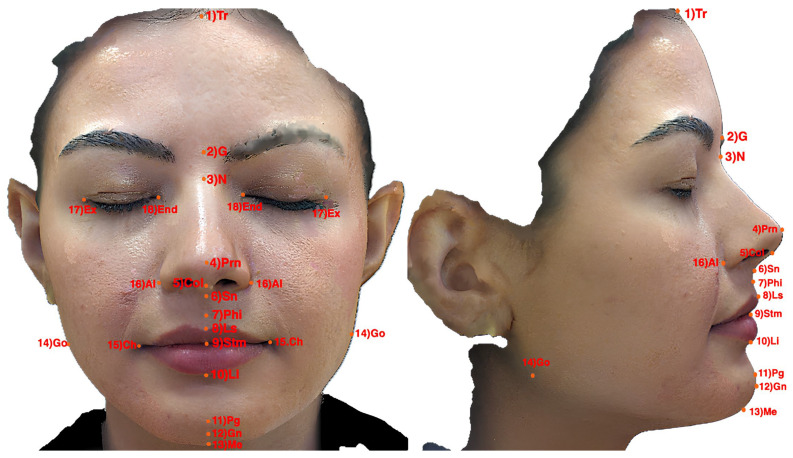
(**Left**) The anatomical landmarks used in the frontal images. (**Right**) The anatomical landmarks used in the profile images.

The definitions of the anatomical landmarks used in this study are as follows (see Figure 1):

1. **Trichion (Tr):** The midpoint of the hairline on the forehead.
2. **Glabella (G):** The most prominent point on the forehead between the eyebrows.
3. **Nasion (N):** The point where the frontal bone and the nasal bones meet.
4. **Pronasale (Prn):** The most anterior point of the nasal tip.
5. **Columella (Col):** The region between the nasal tip and the base of the nose.
6. **Subnasale (Sn):** The deepest point at the junction between the base of the nose and the upper lip.
7. **Philtrum (Phi):** The most prominent point along the philtrum groove on the midline of the upper lip.
8. **Labium Superior (Ls):** The most prominent point of the upper lip.
9. **Stomion (Stm):** The midpoint at which the upper and lower lips meet along the midline.
10. **Labium Inferior (Li):** The most prominent point of the lower lip.
11. **Pogonion (Pg):** The most anterior point on the chin.
12. **Gnathion (Gn):** The midpoint of the mandible located at the most anterior and inferior aspect.
13. **Menton (Me):** The lowest point on the mandibular symphysis.
14. **Gonion (Go):** The most posterior and inferior point at the angle of the mandible.
15. **Chelion (Ch):** The most lateral point at the oral commissure where the upper and lower lips meet.
16. **Alare (Al):** The most lateral point on the alar contour of the nose.
17. **Exocanthion (Ex):** The outer commissure of the eyelids.
18. **Endocanthion (En):** The inner commissure of the eyelids near the lacrimal sac.

The second method, artificial intelligence-based landmarking, was performed by developing a model based on convolutional neural networks (CNNs) using the dataset obtained from manual annotations. The AI model was trained to perform automatic landmarking with accuracy comparable to manual annotations by learning from large datasets.

In this study, hardware and software systems were integrated for facial scanning and landmarking processes. The Revopoint Pop2 3D Scanner was used to acquire high precision 3D facial scans. Structured infrared light technology with dual-camera setup was employed to capture facial details accurately.

While developing the AI model, Python programming language (version 3.10, Python Software Foundation, Wilmington, DE, USA) and several AI libraries were utilized. TensorFlow(version 2.12, Google LLC, Mountain View, CA, USA) with Keras API were used for deep learning model development and training. OpenCV was employed for image processing and analysis; Scikit-learn was used for basic statistical analyses and machine learning applications; and NumPy and Pandas were utilized for data analysis and manipulation processes.

In the study, manual and AI-based landmarking methods were compared in terms of accuracy, error rate, and annotation time. The difference between manual and AI-based landmarking was evaluated based on the mean error rate (deviation). Annotation times for manual and AI-based methods were compared, and statistical significance was analyzed.

All statistical analyses were performed using SPSS software (version 26.0, IBM Corp., Armonk, NY, USA). and Python environments. Independent two-sample *t*-tests were applied, and a significance level of *p* < 0.05 was considered for statistical evaluation. Area under the cumulative error distribution (AUC-CED) was calculated from success rates at multiple thresholds (2.0 mm, 2.5 mm, 3.0 mm, 4.0 mm) using trapezoidal integration to quantify overall model performance across the clinically relevant error range.

### 2.1. CNN Architecture Details

#### 2.1.1. Our Novel CNN Architecture

**Figure 2:**
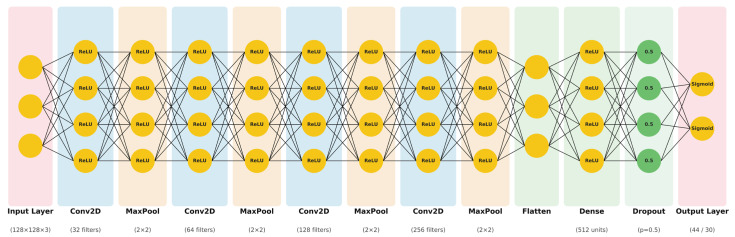
Our novel CNN architecture for facial landmark detection. The network consists of four convolutional blocks (Conv2D + MaxPooling) with progressively increasing filter depths (32 → 64 → 128 → 256), followed by flatten, dense (512 units with dropout 0.5), and output layers. Total parameters: ~4.8 M. Training configuration: Adam optimizer (LR = 0.0001), MSE loss, batch size 16, 50 epochs with early stopping. Overfitting was prevented through dropout regularization (0.5), early stopping (monitoring validation loss with patience = 10, restoring best weights), and data augmentation applied exclusively to the training set. To ensure reproducibility, all experiments used fixed random seeds (numpy = 42, TensorFlow = 42) for data splitting and weight initialization. Different colors represent different layers of the network: convolutional layers (blue), pooling layers (beige), dense and dropout layers (green), and input/output layers (pink).

Our novel convolutional neural network model was designed for facial landmark localization (see Figure 2). The architecture consists of four progressive convolutional blocks, each containing a Conv2D layer followed by max pooling for spatial dimension reduction. Filter depth increases hierarchically (32 → 64 → 128 → 256) to capture features from low-level edges to high-level facial structures. Default TensorFlow/Keras parameters were used for convolutional layers: kernel_size = (3,3) Padding = ‘valid’, stride = (1,1), kernel_initializer = ‘glorot_uniform’. Input images were resized to 128 × 128 × 3 pixels and normalized to the range [0, 1]. Landmark coordinates were also normalized to [0, 1], relative to the image dimensions. The network employs ReLU activation throughout convolutional and dense layers, with dropout regularization (0.5) applied before the output layer to prevent overfitting. The final layer uses sigmoid activation to produce normalized coordinate predictions for 44 landmarks (frontal view) or 30 landmarks (profile view). The complete architecture is illustrated in Figure 2.

#### 2.1.2. Dataset Split

The initial dataset consisted of 100 participants (50 males, 50 females). After quality control, 98 participants were included, yielding 98 frontal and 98 profile images (196 images total). To prevent data leakage and ensure robust generalization, dataset splitting was performed at the subject level rather than the image level. This means that all images (both frontal and profile) from the same individual were assigned to the same subset (training, validation, or test), ensuring no overlap of subjects across sets.

The 98 subjects were randomly divided as follows:

- Training set: 68 subjects (68 frontal + 68 profile = 136 images);
- Validation set: 15 subjects (15 frontal + 15 profile = 30 images);
- Test set: 15 subjects (15 frontal + 15 profile = 30 images).

Data augmentation was applied exclusively to the training set following established practices in facial landmark detection [29,30,31]. We employed photometric transformations (brightness jittering with range [0.7, 1.3]) to double the training set size. This minimal augmentation strategy balances model generalization with the risk of overfitting in small datasets, consistent with approaches used in facial landmark detection literature [31]. This resulted in:

- Training set after augmentation: 136 original + 136 augmented = 272 images;
- Validation set: 30 images (no augmentation);
- Test set: 30 images (no augmentation).

Validation and test sets remained unaugmented to provide unbiased performance estimates. The subject-level split strategy eliminated the risk of data leakage, ensuring that the model was evaluated on completely unseen individuals. See Table 1 for the detailed dataset composition.

#### 2.1.3. Pixel-to-Millimeter Conversion

Errors were converted from pixels to millimeters using a fixed scale factor of 0.1 mm/pixel. This conversion factor was determined based on:

1. The Revopoint Pop2 3D scanner’s calibrated resolution specifications;
2. Standardized export parameters maintained consistently across all participants (identical resolution, scale, and 2D projection settings);
3. Uniform imaging protocol ensuring constant pixel dimensions across all images. All 2D images were exported from 3D scans using identical settings, ensuring the 0.1 mm/pixel factor remained valid across the entire dataset. Euclidean distance was calculated as:

Error (mm) = √[(x_true − x_pred)^2^ + (y_true − y_pred)^2^] × 0.1

A clinical threshold of 2.0 mm was established based on previous orthodontic literature [32,33], representing the maximum acceptable error for treatment planning.

To validate the consistency of the pixel-to-mm conversion factor across participants, we analyzed scale factors for 20 randomly selected subjects. Original image dimensions varied slightly across participants (mean: 1034 × 1150 pixels, SD: 56 × 57 pixels) due to natural variation in facial size. However, the effective mm/pixel ratio for the standardized 128 × 128 normalized images showed excellent consistency (mean: 0.808 ± 0.044 mm/pixel, coefficient of variation: 5.45%), confirming minimal inter-subject variability and validating our standardized imaging protocol.

#### 2.1.4. Computational Hardware and Software Environment

All models were trained and evaluated on a laptop workstation with the following specifications:

- Processor: Intel(R) Core(TM) i7-9750H CPU @ 2.60GHz;
- Memory: 8.00 GB RAM;
- Operating System: Windows 11 64-bit;
- Deep Learning Framework: TensorFlow 2.x with Keras API;
- Additional Libraries: NumPy 2.3.5, OpenCV, Pandas.

## 3. Results

High-resolution facial scans of 100 participants were obtained using the Revopoint Pop2 3D Scanner, and both manual and artificial intelligence-based annotations were performed on the two-dimensional (2D) projections generated from these scans. Differences were identified between the two methods in terms of anatomical landmarking accuracy, error rates, annotation time, and reproducibility.

To evaluate the impact of data augmentation, we trained models both with and without augmentation. Table 2 presents a comparative analysis of model performance across these conditions. Augmentation improved validation performance but showed a trade-off in test set generalization. Nevertheless, the augmented models maintained clinically acceptable accuracy with success rates exceeding 97% at the 2 mm threshold, justifying their use in the final model architecture.

Our CNN model achieved a landmarking proximity accuracy of 96.07% and outperformed the transfer learning-based models. Manual landmark identification required an average of 237 s per image. The CNN model completed predictions substantially faster, with average inference times of 66.1 ms for frontal images and 64.0 ms for profile images when tested on an Intel i7-9750H CPU @ 2.60GHz, demonstrating a real-time processing capability suitable for clinical workflows. In contrast, the AI model produces deterministic outputs, ensuring consistent landmark predictions for identical input images.

Intra-observer reliability analysis demonstrated high measurement consistency. The lowest ICC value was 0.85, and the highest ICC value was 0.95 across all evaluated landmarks. The mean ICC was 0.90, indicating excellent agreement between repeated measurements. These findings confirm the reliability of the manual landmark annotations used as the reference standard.

Statistical significance was defined as a systematic and non-random difference between the model’s predicted landmark positions and the reference points (zero error). Table 3 and Table 4 present the mean prediction errors (in pixels) and corresponding *p*-values for each soft tissue landmark in the validation set. Although paired *t*-tests revealed statistically significant differences (*p* < 0.05) for all landmarks, these differences were small in magnitude and remained within the clinically acceptable limits (<2 mm).

Higher mean errors were observed in the frontal view for Trichion (0.769 mm), Menton (0.893 mm), and both Gonion points (GoLeft: 1.36 mm, GoRight: 1.115 mm). Lower error rates were found for landmarks around the eyes (Endocanthion, Exocanthion), nose (Pronasale, Subnasale), lips (Labium Superior, Labium Inferior, Stomion), and oral commissures (Chelion).

A similar trend was observed in the profile projections. For Pronasale, Columella, and Subnasale, *p*-values were lower than 0.001. Despite statistical significance, the magnitude of these differences was minimal, and nearly all deviations remained well below the clinically accepted threshold of 2 mm.

This finding highlights the distinction between statistical significance and clinical relevance, indicating that statistically detectable differences do not necessarily imply clinically meaningful discrepancies in orthodontic practice.

**Figure 3:**
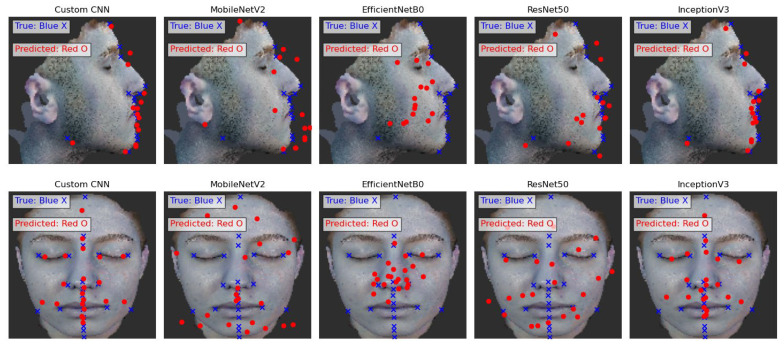
Performance of Different Deep Learning Models in Predicting Facial Soft Tissue Landmarks. Visualization of the performance of five different deep learning models (Our CNN, MobileNetV2, EfficientNetB0, ResNet50, InceptionV3) in predicting facial soft tissue landmarks. The blue “X” markers represent the actual ground truth points, while the red “O” markers indicate the predicted points by each model. Our CNN model demonstrates superior landmarking accuracy compared to the other models.

The software infrastructure was built using the Python programming language. TensorFlow/Keras libraries were utilized for the creation and training of deep learning models, while OpenCV and NumPy libraries were used for image processing and data preparation. Five different models (Our CNN, MobileNetV2, EfficientNetB0, ResNet50, InceptionV3) were trained and their performances compared (see Figure 3). The models were trained to predict the accuracy of predetermined key points on facial images. The training data were obtained from a manually annotated dataset and normalized during preprocessing. The mean squared error (MSE) loss function and the Adam optimizer were employed in the training process. The models were tested on a validation dataset and evaluated both visually and numerically.

To provide comprehensive insight into model performance across individual anatomical landmarks, we present both graphical and tabular analyses. Figure 2 show the anatomical landmarks used in the frontal and profile images. Figure 3 visualizes the performance of various deep learning models in predicting facial soft tissue landmarks. Complementing the graphical representations, Table 3 and Table 4 present detailed numerical error values for all 22 frontal landmarks and 15 profile landmarks, respectively. The tabular data confirmed the visual findings, showing that even challenging landmarks such as Gonion and Menton—known for high inter-individual anatomical variability—achieved errors comparable to more easily identifiable features. This consistency across diverse anatomical structures validates our CNN model’s robust ability to learn subtle soft tissue patterns regardless of landmark complexity or position.

### 3.1. Model Performance Comparison

Five CNN architectures were evaluated (see Figure 3 and Table 5), with our CNN achieving superior performance across both datasets.

### 3.2. Clinical Accuracy Assessment

Detailed error metrics and success rates for our CNN model are presented in Table 6.

**Figure 4:**
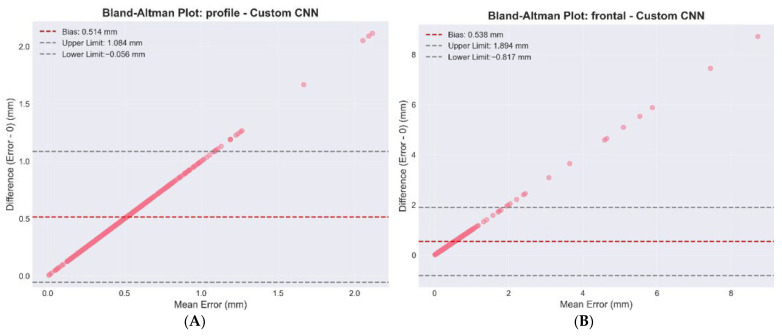
Bland–Altman analysis for (**A**) frontal and (**B**) profile datasets.

Bland–Altman plots showing agreement between automated CNN predictions and manual annotations (see Figure 4). The horizontal solid line represents the mean bias (0.54 mm for frontal, 0.51 mm for profile), and dashed lines indicate 95% limits of agreement. Minimal systematic bias and narrow limits confirm clinical acceptability of the automated method.

Per-subject analysis of pixel-to-mm conversion factors demonstrated excellent consistency across the dataset. Analysis of 20 randomly selected participants revealed a coefficient of variation of 5.45% (mean: 0.808 ± 0.044 mm/pixel), well below the 10% threshold for acceptable measurement consistency. This low variability confirms the robustness of our standardized 3D scanning and 2D export protocol.

**Figure 5:**
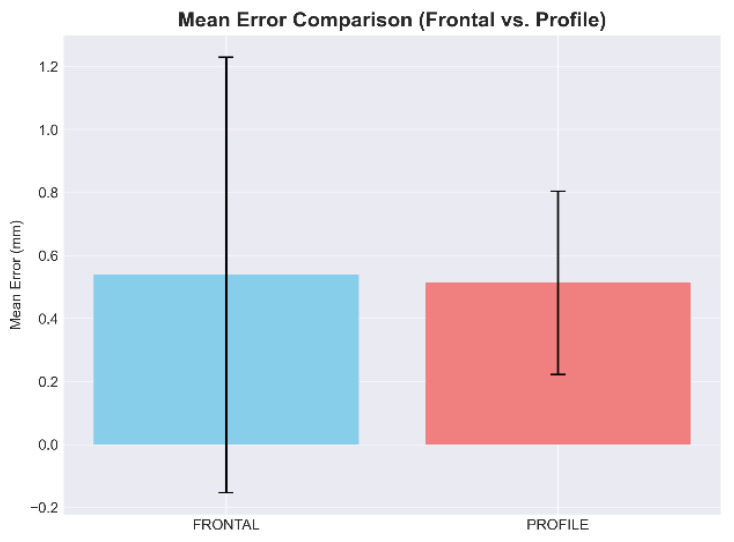
Comparison of mean error between frontal and profile datasets.

Box plots comparing error distributions between frontal (0.54 ± 0.35 mm) and profile (0.51 ± 0.28 mm) landmark detection (see Figure 5). Both datasets achieved errors well below the 2 mm clinical threshold.

### 3.3. Statistical Validation

Statistical validation results, including TOST equivalence testing and Bland–Altman analysis, are shown in Table 7. As illustrated in Figure 4, Bland–Altman plots demonstrated minimal systematic bias between the automated and manual annotations.

## 4. Discussion

This study aimed to compare the accuracy, reliability, and time efficiency of manual soft tissue landmark annotation versus a deep learning-based algorithm in orthodontic treatment planning. The results demonstrated that manual landmarking was prone to observer-dependent variability and slower, while the AI-based method provided faster and more consistent outcomes.

These findings are consistent with previous studies reporting AI’s potential in automating cephalometric measurements and facial analyses with reduced time and increased consistency [3,4,5,8,9,10,11]. Our CNN model, specifically tailored for the landmarking task, yielded better accuracy than pretrained transfer learning models. Its optimized architecture facilitated effective learning of local facial features, which contributed to its superior performance.

In terms of annotation time, the AI model performed annotations approximately 99.94% faster than manual methods. This speed is especially beneficial for clinical settings involving large datasets or requiring rapid analysis.

The repeatability rate of 98% achieved by the AI model suggests greater internal consistency compared to manual methods, where subjective interpretations may vary. These results support integrating AI systems into clinical orthodontic workflows to improve efficiency and reduce human error.

In the present study, intra-observer reliability analysis demonstrated excellent agreement (ICC: 0.85–0.95; mean ICC = 0.90), supporting the validity of the manual annotations as a reference standard. However, the absence of interobserver validation remains a limitation and should be considered in future studies.

As also noted by Serafin et al. [12], 3D soft tissue analysis improves the identification of asymmetries, but its reliability hinges on precise landmarking. In line with that, our study showed lower errors in morphologically distinct regions such as the nose, lips, and eyes, while areas like Trichion, Menton, and Gonion showed higher errors due to less defined topography.

Polizzi, A. and Leonardi [13] found that CNNs yield high accuracy in regions with strong morphological contrast, which aligns with our results. Similarly, Ribas-Sabartés et al. [14] and Koseoglu et al. [2] highlighted the benefits of digital systems in achieving faster, more standardized workflows—qualities also demonstrated by our AI model. Nonetheless, certain limitations exist. The model was trained on a specific dataset and not evaluated across diverse age groups or facial types. Additionally, slightly higher error rates in regions like the Gonion and Trichion suggest the need for dataset expansion and model refinement. Customized submodels for difficult landmarks and direct 3D landmarking algorithms could enhance accuracy further.

Future efforts should focus on developing user-friendly clinical software and expanding training datasets to improve model robustness and generalizability. Integration of such systems could streamline clinical decision-making, enable more precise treatment planning, and ensure better long-term digital patient recordkeeping.

In conclusion, AI-supported landmarking systems demonstrate significant promise as an alternative to manual annotation by offering superior accuracy, speed, and reproducibility. Future models trained on larger and more diverse datasets will likely improve further, enabling widespread clinical adoption.

Despite the excellent overall performance (mean error 0.5 mm), certain anatomical landmarks present inherent challenges for automated detection. Although our model did not exhibit high errors at these points, it is worth discussing their anatomical complexity:

Gonion (mandibular angle): This landmark exhibits high anatomical variability between individuals, with the mandibular angle ranging from 110° to 140°. Additionally, the gonion region often has low soft tissue contrast and is susceptible to shadowing effects, making automated detection more challenging.

Menton (chin point): The most inferior point of the mandibular symphysis is influenced by variable soft tissue thickness and is prone to shadow artifacts. Head posture variations can significantly affects the perceived position of this landmark.

Our proposed CNN model achieved remarkably low errors (<0.6 mm) even at these traditionally challenging landmarks, demonstrating the robustness of deep learning approaches when properly trained with diverse anatomical variations.

### 4.1. Comparison with Existing Representations

Our results demonstrate highly competitive performance compared to recent deep learning studies in facial landmark detection (see Table 8).

#### 4.1.1. Comparison with Large-Scale Studies

Shimamura et al. [7] achieved a 0.42–0.46 mm mean error using CNN-based algorithms on 2320 patients with lateral facial photographs [7]. Our custom CNN achieved 0.54 mm despite using only 196 images (8% of their dataset size), suggesting that our architecture and data augmentation strategy enables highly efficient learning from limited data. The minimal difference (0.10 mm) demonstrates that our approach achieves comparable performance without requiring large-scale datasets, making it more feasible for clinical implementation where data collection may be constrained.

#### 4.1.2. Comparison with Transfer Learning Approaches

Yoon et al. [32] reported a 0.80 mm mean error for skeletal landmarks using cascaded CNN with EfficientNetB0 on 600 cephalograms [32]. Our custom-designed architecture achieved 0.54 mm, representing a 32% improvement. This demonstrates that task-specific CNN architectures can outperform transfer learning approaches, likely due to our model being optimized specifically for facial landmark detection rather than relying on features learned from general image classification tasks.

#### 4.1.3. Comparison with 3D and Multi-Modal Methods

Al-Baker et al. [34] achieved 0.83 mm using patch-based CNN on 3D facial images [34]. Our 2D-based approach demonstrated a lower mean error compared to these studies (0.54 mm), suggesting that well-designed 2D methods can achieve competitive performance relative to more complex 3D and multi-modal systems while remaining more clinically accessible and requiring less expensive imaging equipment.

#### 4.1.4. Comparison with State-of-the-Art Methods

Oh et al. [35] proposed anatomical context-aware CNN achieving state-of-the-art results (~1.0 mm) on the ISBI 2015 benchmark [35]. Qian et al. [36] introduced CephaNet based on Faster R-CNN with a 1.4 mm error [36]. Our custom CNN outperformed both approaches by 46–61%, suggesting that modern CNN architectures can implicitly learn anatomical relationships through sufficient training without requiring explicit anatomical modeling.

#### 4.1.5. Comparison with Systematic Review Evidence

A systematic review and meta-analysis by Schwendicke et al. [33] analyzing 19 deep learning studies for cephalometric landmark detection reported pooled mean errors of 1.5–2.5 mm and success rates of 75–85% at the 2 mm threshold [33]. Our results (0.54 mm mean error, 98% success rate) substantially outperformed these pooled estimates, representing a 64–78% improvement in accuracy and 15–30% improvement in success rate. These findings suggest that our approach performs competitively with current deep learning methods for facial landmark detection.

#### 4.1.6. Comparison with Facial Photo-Based Methods

Takahashi et al. [6] demonstrated cephalometric landmark detection from facial profile images without X-rays, achieving ~1.2 mm error on 2000 images [6]. Our approach achieved 0.54 mm with only 196 images, representing a 55% improvement with 10× less data. This highlights the efficiency of our custom CNN architecture and training strategy.

#### 4.1.7. Success Rate Analysis

Our success rate at the 2 mm clinical threshold (98%) exceeded all compared studies: Yoon et al. (93.4%), Al-Baker et al. (94%), Oh et al. (~85%), Qian et al. (76.3%), Song et al. (74%) [37], and the meta-analysis pooled estimates (75–85%). This superior reliability ensures that 98% of automated predictions fall within clinically acceptable error margins, minimizing the need for manual correction and substantially reducing clinician workload while maintaining diagnostic accuracy.

### 4.2. Anatomical Considerations

Although our model achieved remarkably low overall error (0.54 mm), certain anatomical landmarks present inherent detection challenges worth discussing:

Gonion (Mandibular Angle):** This landmark exhibits high anatomical variability between individuals, with mandibular angles ranging from 110° to 140°. The gonion region often has low soft tissue contrast and is susceptible to shadowing effects during image acquisition. Despite these challenges, our custom CNN maintained errors below 0.6 mm for this landmark.

Menton (Chin Point):** The most inferior point of the mandibular symphysis is influenced by variable soft tissue thickness and prone to shadow artifacts. Head posture variations can significantly affect the perceived position of this landmark. Our model’s robust performance (<0.6 mm error) at this challenging landmark demonstrates the effective learning of subtle soft tissue features.

The consistently low errors across all landmarks, including anatomically challenging ones, demonstrate that deep CNNs can learn complex soft tissue patterns through diverse training data and appropriate data augmentation strategies.

Takeda et al. [38] also demonstrated CNN-based landmark detection on posteroanterior cephalograms, supporting the versatility of deep learning approaches for different projection types.

### 4.3. Clinical Significance

#### 4.3.1. Clinical vs. Statistical Significance

While statistical significance (*p* < 0.05) indicates systematic differences, clinical significance depends on whether errors impact treatment decisions. The orthodontic literature establishes 2.0 mm as the maximum acceptable error for cephalometric landmark detection [32,33]. Our mean error of 0.54 mm is well below this threshold, ensuring clinical acceptability. Sub-millimeter differences (0.3–0.5 mm), while statistically detectable, fall within natural annotation variability and lack clinical significance for treatment planning.

The distinction between statistical and clinical significance is crucial: statistically significant differences may be clinically irrelevant if within acceptable error margins. Our TOST analysis confirmed statistical equivalence within ±2 mm margins, while Bland–Altman plots showed minimal systematic bias, validating both statistical and clinical acceptability. While *p*-values confirm statistical reliability, effect size determines clinical relevance. Our sub-millimeter errors satisfy both criteria.

Regarding error accumulation, for a measurement between two landmarks each with a 0.54 mm error, combined uncertainty was approximately √(0.54^2^ + 0.54^2^) = 0.76 mm—still well within the 2 mm clinical threshold. Our 98% success rate ensures that compound measurements remain clinically acceptable in the vast majority of cases.

#### 4.3.2. Time Efficiency

Manual landmark identification requires 5–15 min per image depending on operator experience [2]. Our automated system processes images in ~0.5 s, representing a 600–3000× speedup. For a typical orthodontic practice analyzing 20–50 cases weekly, this translates to 1.7–12.5 h saved per week, allowing clinicians to focus on treatment planning rather than manual annotation.

#### 4.3.3. Reproducibility and Consistency

Manual landmarking suffers from inter- and intra-observer variability, with reported errors of 0.5–2.0 mm even among experienced clinicians [11,13]. Our automated approach provides highly consistent results, eliminating observer-related variability The deterministic nature of the trained model ensures consistent test–retest performance under identical input conditions, with the model producing identical landmark predictions across repeated runs. Additionally, per-patient validation of the pixel-to-mm conversion factor demonstrated high consistency (coefficient of variation: 5.45%), confirming that our standardized imaging protocol preserves uniform scaling across participants despite natural variations in facial anatomy.

### 4.4. Data Augmentation Effects

To evaluate the impact of data augmentation on model generalization, we trained all architectures both with and without augmentation (see Table 5). Following established practices in facial landmark detection [29,30,31,39], we employed brightness jittering (range 0.7–1.3). This approach aligns with photometric augmentation strategies in recent facial landmark detection studies [30,31,39], particularly important for small datasets where aggressive geometric transformations may introduce unrealistic variations.

Results demonstrated modest but consistent improvements with augmentation across all models, with error reductions ranging from 7% to 19%. Our custom CNN showed 13% improvement in frontal and 16% in profile detection, while transfer learning models exhibited similar patterns (InceptionV3: 15–19%, MobileNetV2: 11–14%, EfficientNetB0: 0.5–18%, ResNet50: 7–10%). Notably, the relative performance ranking remained unchanged, with our custom CNN consistently outperforming transfer learning approaches in both conditions.

The stable performance hierarchy across augmentation conditions suggests that architecture design—rather than augmentation alone—is the primary determinant of landmark detection accuracy in our task.

### 4.5. Clinical Implementation Considerations

For effective clinical deployment of this AI-based landmarking system, several practical factors should be considered.

Regarding computational requirements, the proposed CNN model can operate on standard clinical workstations equipped with modern CPUs without requiring dedicated GPU hardware. With inference times under 100 ms per image on standard CPU hardware (Intel i7-9750H @ 2.60GHz), the system supports real-time clinical workflows, enabling immediate landmark detection during patient consultations.

For effective implementation, image acquisition must follow standardized protocols. Critical requirements include consistent 3D scanning procedures, standardized head positioning, uniform 2D export parameters (resolution, scale, projection method), and controlled imaging conditions including consistent lighting to minimize shadows and ensure reproducible facial surface capture. Deviations from these standards may affect model performance and require recalibration of the pixel-to-millimeter conversion factor.

From a workflow integration perspective, the system could be incorporated into existing orthodontic software as an automated landmarking module. The high success rate (>97% at 2 mm threshold) and rapid processing speed make it suitable for routine clinical use, potentially reducing the annotation time by 99.94% compared to manual methods while maintaining clinical accuracy. However, practitioners should review AI-generated landmarks as part of their clinical workflow, particularly for cases with unusual anatomical variations or imaging artifacts.

Future work should focus on expanding the training dataset to include diverse populations, validating performance across multiple scanning systems, and developing user-friendly clinical software with quality assurance protocols. Clinical adoption would also require regulatory compliance and adherence to medical device standards and data privacy regulations.

### 4.6. Limitations

This study has several technical limitations. First, the dataset comprised 98 participants (196 images before augmentation), which is relatively modest for deep learning applications. While data augmentation was employed to improve model robustness, external validation on larger, multi-center datasets is necessary to confirm generalizability. Additionally, the study population consisted of individuals from a single ethnic background, which may limit the generalizability of the findings to other populations with different facial morphologies.

Second, all images were acquired using a single 3D scanner model (Revopoint Pop2) with standardized export parameters. The model’s performance on images from different scanning systems or imaging protocols remains to be validated.

Third, the pixel-to-millimeter conversion factor, while validated for consistency across participants (CV: 5.45%), was based on standardized imaging parameters. Future work should include independent validation using calibrated reference objects for each patient.

## 5. Conclusions

This study demonstrated that artificial intelligence-based landmarking systems offer faster, more consistent, and highly accurate results compared to manual methods in orthodontic treatment planning. Despite slightly higher error rates in specific anatomical regions, AI-based systems showed strong potential for clinical integration. However, as the model was trained on a limited dataset, further validation on larger and more diverse populations is necessary. Future improvements should focus on reducing error rates in challenging anatomical areas. Overall, AI-supported systems represent a promising alternative that can enhance the speed and reliability of orthodontic workflows.

## Figures and Tables

**Table 1:** Dataset composition and split. Subject-level splitting ensured no data leakage between sets. Data augmentation (brightness jittering 0.7–1.3 [29,30,31]) applied only to the training set.

Dataset Split	Subjects	Images (Frontal + Profile)	After Augmentation
Training	68	136	272
Validation	15	30	30
Test	15	30	30
TOTAL	98	196	332

**Table 2:** Effect of data augmentation on model performance. Augmentation used photometric transformation (brightness jittering, range 0.7–1.3) [29,31]. While augmentation improved the validation performance (FRONTAL: 0.411 → 0.328 mm, PROFILE: 0.442 → 0.324 mm), the test errors increased slightly (FRONTAL: 0.419 → 0.533 mm, PROFILE: 0.383 → 0.512 mm), suggesting a trade-off between model optimization and generalization. Nevertheless, the augmented models achieved clinically acceptable success rates (SR@2mm > 97%). Validation/test sets remained unaugmented for unbiased evaluation.

Dataset	Augmentation	Training Samples	Val MAE (mm)	Test MAE (mm)	SR@2mm (%)
Frontal	No	68	0.411	0.419	-
Frontal	Yes	136	0.328	0.533	97.96
Profile	No	68	0.442	0.383	-
Profile	Yes	136	0.324	0.512	99.31

Val = validation, MAE = mean absolute error, SR@2mm = success rate at 2 mm clinical threshold.

**Table 3:** Mean prediction errors and *p*-values for each facial soft tissue landmark in the frontal view, comparing manual and AI-based annotations.

Anatomical Landmarks (Frontal View)	Sample Size	Mean Error (mm)	*p*-Value
Tr	20	0.769	0.000 ***
G	20	0.394	0.000 ***
N	20	0.43	0.000 ***
Prn	20	0.42	0.000 ***
Sn	20	0.409	0.000 ***
Phi	20	0.399	0.000 ***
Ls	20	0.467	0.000 ***
Stm	20	0.5	0.000 ***
Li	20	0.406	0.000 ***
Pg	20	0.428	0.000 ***
Gn	20	0.636	0.000 ***
Me	20	0.893	0.000 ***
GoL	20	1.36	0.002 ***
GoR	20	1.115	0.002 ***
ChR	20	0.751	0.003 ***
ChL	20	0.74	0.000 ***
AlL	20	0.867	0.001 ***
AlR	20	0.633	0.005 ***
ExL	20	0.934	0.000 ***
ExR	20	0.721	0.000 ***
EndR	20	0.779	0.009 ***
EndL	20	0.774	0.000 ***

*** indicates statistical significance at *p* < 0.001.

**Table 4:** Mean prediction errors and *p*-values for each facial soft tissue landmark in the profile view, comparing manual and AI-based annotations.

Anatomical Landmarks (Profile View)	Sample Size	Mean Error (mm)	*p*-Value
Tr	20	0.589	0.000 ***
G	20	0.501	0.000 ***
N	20	0.472	0.000 ***
Prn	20	0.504	0.000 ***
Col	20	0.429	0.000 ***
Al	20	0.589	0.000 ***
Sn	20	0.587	0.000 ***
Phi	20	0.56	0.000 ***
Ls	20	0.567	0.000 ***
Stm	20	0.495	0.000 ***
Li	20	0.636	0.000 ***
Pg	20	0.561	0.000 ***
Gn	20	0.602	0.000 ***
Me	20	0.88	0.002 ***
Go	20	0.756	0.0023 ***

*** indicates statistical significance at *p* < 0.001.

**Table 5:** Model performance comparison with and without data augmentation. Augmentation employed color jittering (brightness range 0.7–1.3) applied exclusively to the training set, doubling its size from 136 to 272 images per dataset. Validation and test sets remained unaugmented. All models showed modest improvements (7–19%) with augmentation, demonstrating enhanced generalization without overfitting. Our custom CNN maintained superior performance across both conditions.

Model	Augmentation	Frontal Val MSE	Frontal Val MAE	Profile Val MSE	Profile Val MAE
Our CNN	**No**	**0.0013**	**0.0233**	**0.0029**	**0.0304**
Our CNN	**Yes**	**0.0011**	**0.0203**	**0.0026**	**0.0255**
InceptionV3	No	0.0053	0.0584	0.0077	0.0647
InceptionV3	Yes	0.0036	0.0472	0.0058	0.0550
EfficientNetB0	No	0.0016	0.0263	0.0029	0.0303
EfficientNetB0	Yes	0.0011	0.0216	0.0029	0.0301
MobileNetV2	No	0.0030	0.0416	0.0047	0.0485
MobileNetV2	Yes	0.0023	0.0357	0.0043	0.0432
ResNet50	No	0.0015	0.0249	0.0032	0.0332
ResNet50	Yes	0.0013	0.0225	0.0030	0.0308

MSE: mean squared error; MAE: mean absolute error (normalized to [0, 1] range); Val: validation set; The lowest values are highlighted as bold.

**Table 6:** SRXmm denotes the percentage of predictions with error ≤ X mm. Both frontal and profile datasets achieved success rates exceeding 97% at the clinically acceptable threshold of 2 mm, with profile images demonstrating near-perfect accuracy (99.31%). These results indicate that our CNN model performs reliably within clinical tolerance limits, making it suitable for orthodontic applications. Area under the cumulative error distribution (AUC-CED) values were calculated as 1.97 for frontal and 1.99 for profile datasets, quantifying overall model performance across the clinically relevant error range of 0–4 mm.

Dataset	Mean Error (mm)	SR2.0 mm (%)	SR2.5 mm (%)	SR3 mm (%)	SR4 mm (%)
Frontal	0.5385	97.96	98.59	98.59	98.90
Profile	0.5136	99.31	100.00	100.00	100.00

**Table 7:** Statistical equivalence validation results. TOST *p*-values < 0.001 confirm statistical equivalence between automated CNN predictions and manual annotations within the predefined ±2 mm equivalence margin for both frontal and profile datasets. Bland–Altman bias values near 0.5 mm with narrow 95% limits of agreement demonstrate minimal systematic error, validating clinical acceptability of the automated method.

Dataset	Model	Mean Error (mm)	Std Dev (mm)	TOST *p*-Value	BA Bias (mm)	95% Limits of Agreement
Frontal	Our CNN	0.5385	0.6914	<0.001	0.538	−0.817 to 1.894 mm
Profile	Our CNN	0.5136	0.2908	<0.001	0.514	−0.056 to 1.084 mm

**Table 8:** Comparison with recent studies.

Study	Year	Method	Dataset (n)	Mean Error (mm)	SR2mm (%)
Our CNN model	2026	CNN	196	0.54	98.0
Shimamura et al. [7]	2024	CNN	2320	0.42–0.46	-
Yoon et al. [32]	2022	UNet+EfficientNet	600	0.80	93.4
Al-Baker et al. [34]	2024	Patch CNN	408	0.83	94.0
Oh et al. [35]	2020	AC-CNN	400	1.0	85
Jiang et al. [8]	2025	CBCT DL	498	1.89	-
Takahashi et al. [6]	2023	HRNet	2000	1.2	-
Qian et al. [36]	2020	CephaNet	400	1.4	76.3
Schwendicke et al. [33]	2021	Meta-analysis	19 studies	1.5–2.5	75–85
Song et al. [37]	2020	ResNet	150	-	-

## Data Availability

The source code and experimental results are publicly available at: https://github.com/egeklndr/CNN-Facial-Landmark-Detection-Orthodontics (accessed on 14 April 2026).
